# Metatranscriptomics Reveals the Diversity of the Tick Virome in Northwest China

**DOI:** 10.1128/spectrum.01115-22

**Published:** 2022-10-10

**Authors:** Yunyi Kong, Gang Zhang, Lingling Jiang, Pu Wang, Sinong Zhang, Xiaomin Zheng, Yong Li

**Affiliations:** a Key Laboratory of Ministry of Education for Protection and Utilization of Special Biological Resources in Western China, School of Life Sciences, Ningxia University, Yinchuan, China; b Research Institute for Reproductive Medicine and Genetic Diseases, Affiliated Wuxi Maternity and Child Health Care Hospital of Nanjing Medical University, Wuxi, China; Changchun Veterinary Research Institute

**Keywords:** ticks, tick-borne viruses, metatranscriptomics, phylogenetic analysis

## Abstract

Blood-sucking ticks are obligate parasites and vectors of a variety of human and animal viruses. Some tick-borne viruses have been identified as pathogens of infectious diseases in humans or animals, potentially imposing significant public health burdens and threats to the husbandry industry. Therefore, identifying the profiles of tick-borne viruses will provide valuable information about the evolution and pathogen ecology of tick-borne viruses. In this study, we investigated the viromes of parasitic ticks collected from the body surfaces of herbivores in Xinjiang Uyghur Autonomous Region and Inner Mongolia Autonomous Region of China, two regions in northwest China. By using a metatranscriptomic approach, 17 RNA viruses with high diversity in genomic organization and evolution were identified. Among them, nine are proposed to be novel species. The classified viruses belonged to six viral families, including *Phenuiviridae*, *Rhabdoviridae*, *Peribunyaviridae*, *Lispiviridae*, *Chuviridae*, and *Reoviridae*, and unclassified viruses were also identified. In addition, although some viruses from different sampling locations shared significant similarities, the abundance and diversity of viruses notably varied among the different collection locations. This study demonstrates the diversity of tick-borne viruses in Xinjiang and Inner Mongolia and provides informative data for further study of the evolution and pathogenicity of these RNA viruses.

**IMPORTANCE** Ticks are widely distributed in pastoral areas in northwestern China and act as vectors that carry and transmit a variety of pathogens, especially viruses. Our study revealed the diversity of tick viruses in Xinjiang and Inner Mongolia and uncovered the phylogenetic relationships of some RNA viruses, especially the important zoonotic tick-borne severe fever with thrombocytopenia syndrome virus in Inner Mongolia. These data suggest a complex and diverse evolutionary history and potential ecological factors associated with pathogenic viruses. The pathogenicity of these tick-borne viruses currently remains unclear. Therefore, future research should focus on evaluating the transmissability and pathogenicity of these tick-borne viruses.

## INTRODUCTION

Ticks are the second most important arboviral vector after mosquitoes and are able to transmit a large number of viruses. To date, more than 160 tick-borne viruses (TBVs) that belong to two orders and at least 12 genera in 9 families have been identified (1, 2). Importantly, 20% to 30% of tick-borne viruses are pathogenic to humans or livestock, causing substantial public health concern and challenges for disease prevention and control (3). Multiple TBVs have been reported in China. For instance, tick-borne encephalitis virus (TBEV) in northeast China (4) and Crimean-Congo hemorrhagic fever virus (CCHFV) in Xinjiang Autonomous Region have been reported for a long time, and sporadic outbreaks with fatal cases have occurred for decades (5, 6). Severe fever with thrombocytopenia syndrome virus (SFTSV) is another virus that was first discovered in patients with acute fever and severe thrombocytopenia and who were diagnosed with severe fever with thrombocytopenia syndrome (SFTS) in Henan Province, China, in 2009. The pathogen of SFTS was identified by metagenomic analysis in 2011 and named SFTSV (7, 8). Currently, SFTSV has become a highly pathogenic tick-borne virus that is widespread in Southeast Asia, including China, Japan, South Korea, Myanmar, and Vietnam (9, 10). TBVs also have an economic impact on the husbandry industry. For example, African swine fever virus (ASFV) was first detected on a pig farm in Shenyang, China, and gradually spread to several provinces, causing significant losses to farmers in 2018 (11, 12). Therefore, identification of TBVs in natural environments will provide more accurate surveillance data and allow the prediction of epidemic risks.

Methodologically, metaviromics has been a reliable approach for profiling vector-borne viruses. Next-generation sequencing (NGS) provides nucleic acid information and can help in the identification of viruses in samples of various organisms (13–16). Metaviromics has been employed for organisms with high economic value or of significant public health and safety concern, such as bees and mosquitoes. Indeed, through metaviromics, seven novel viruses have been identified in bee populations in different geographical regions, including two new rhabdoviruses and two new noroviruses in mosquitoes in Australia and five new viruses in *Culex* spp. mosquitoes in Scotland (17–19). Similarly, many novel TBVs have been identified by the NGS approach and metaviromic analyses. For example, several new TBVs were identified in ticks in Hubei and Yunnan Provinces of China, nine unknown viruses were found in Ixodes ricinus in northern Europe, and several TBVs have been identified in Colombia and Trinidad and Tobago in South America (20–24).

Xinjiang and Inner Mongolia are two autonomous regions with vast territories and high richness of wild animals and agricultural resources in China. They are important animal husbandry industry regions in China. More than 40 species of ticks have been reported in China, and 20 species have been reported in Xinjiang and Inner Mongolia (25). Several new pathogenic TBVs, such as Alongshan virus, Songling virus, Tacheng tick virus 1, and Tacheng tick virus 2, have been identified in these areas (26–29). Thus, it is necessary to further identify viruses carried by ticks in these two areas. In this study, we profiled tick-borne viruses in ticks collected from Xinjiang Uyghur Autonomous Region and Inner Mongolia Autonomous Region in China.

## RESULTS

### Diversity and composition of the tick virome.

**FIG 1:**
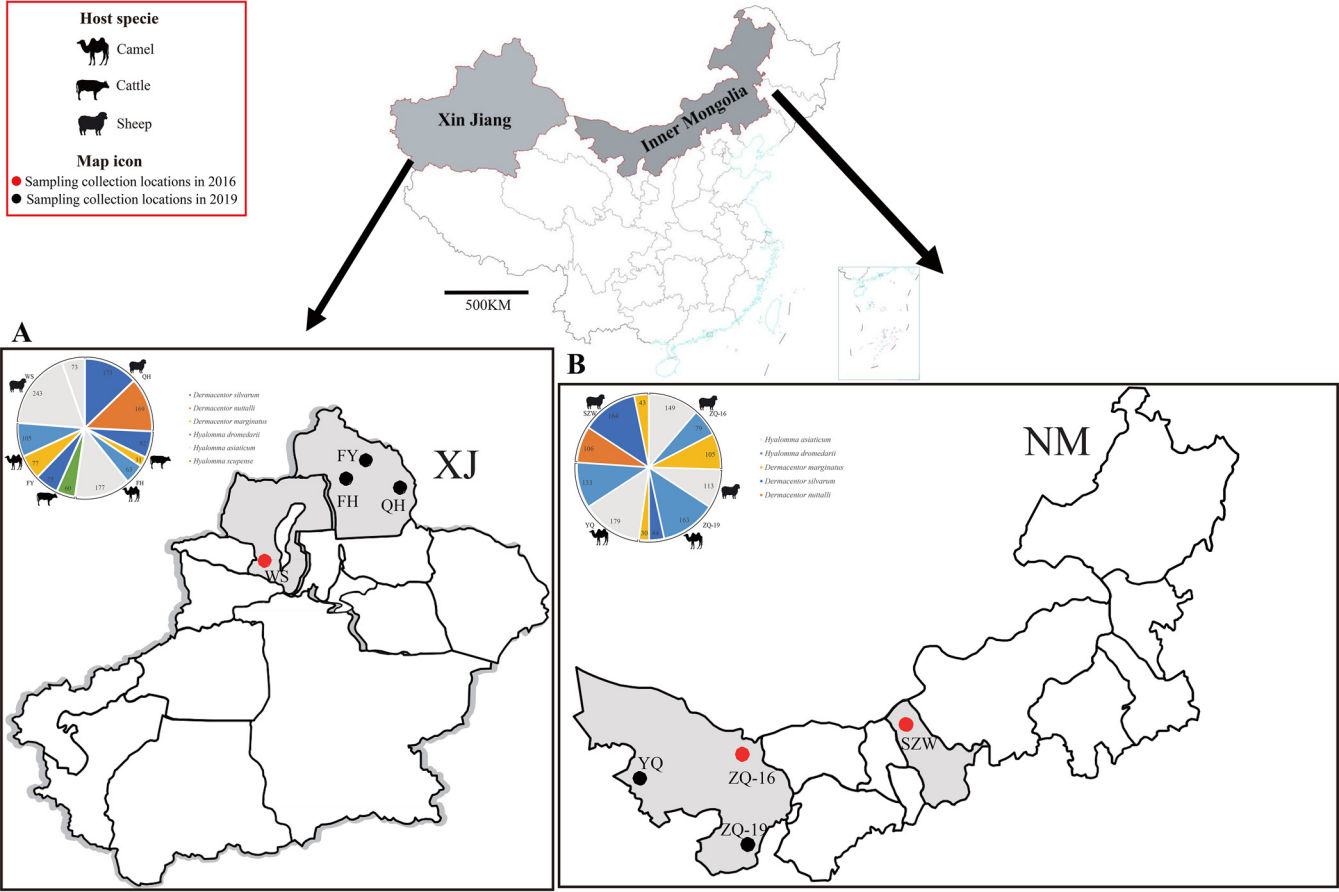
Distribution of tick sampling collection locations and species compositions of ticks from the sampling sites. Species of ticks are represented by different colors (see color legend). Animal icons represent host species. Abbreviations in the pie charts represent tick collection sites. The panels show the sampling locations and tick data in Xinjiang (A) and Inner Mongolia (B). Abbreviations: FY, Fuyun; FH, Fuhai; QH, Qinghe; WS, Wusu; ZQ, Alxa Left Banner; YQ, Alxa Right Banner; SZW, Siziwang Banner.

We characterized eight metatranscriptomic sequencing libraries constructed from 2,636 ticks collected from herbivore livestock (Fig. 1 and Table 1). The total sequencing reads of the eight tick libraries were spliced, and low-quality reads were removed. Overall, 67,286,426 to 125,151,766 clean reads were obtained. These clean reads were then assembled into 11,821 to 999,230 contigs (Table 1). The abundance of viral reads among these eight tick libraries varied greatly, from 0.014% to 0.336% (Table 1).

**TABLE 1:** Overview of the different metatranscriptomic sequencing libraries

Group	Yr of sampling	RNA concn (ng/μL)	No. of reads	No. of clean reads	No. of contigs	Viral read abundance (%)
QH	2019	476	76,014,658	75,177,816	26,764	0.016
FH	2019	515	79,263,006	78,492,508	11,821	0.136
FY	2019	550	71,643,740	71,058,740	24,448	0.077
ZQ19	2019	650	68,013,002	67,286,426	16,344	0.080
YQ	2019	700	67,395,502	66,862,922	24,299	0.336
WS	2016	450	126,915,640	125,151,766	999,230	0.058
ZQ16	2016	610	96,855,258	95,374,312	18,207	0.187
SZW	2016	520	110,445,692	108,096,930	778,112	0.014

**FIG 2:**
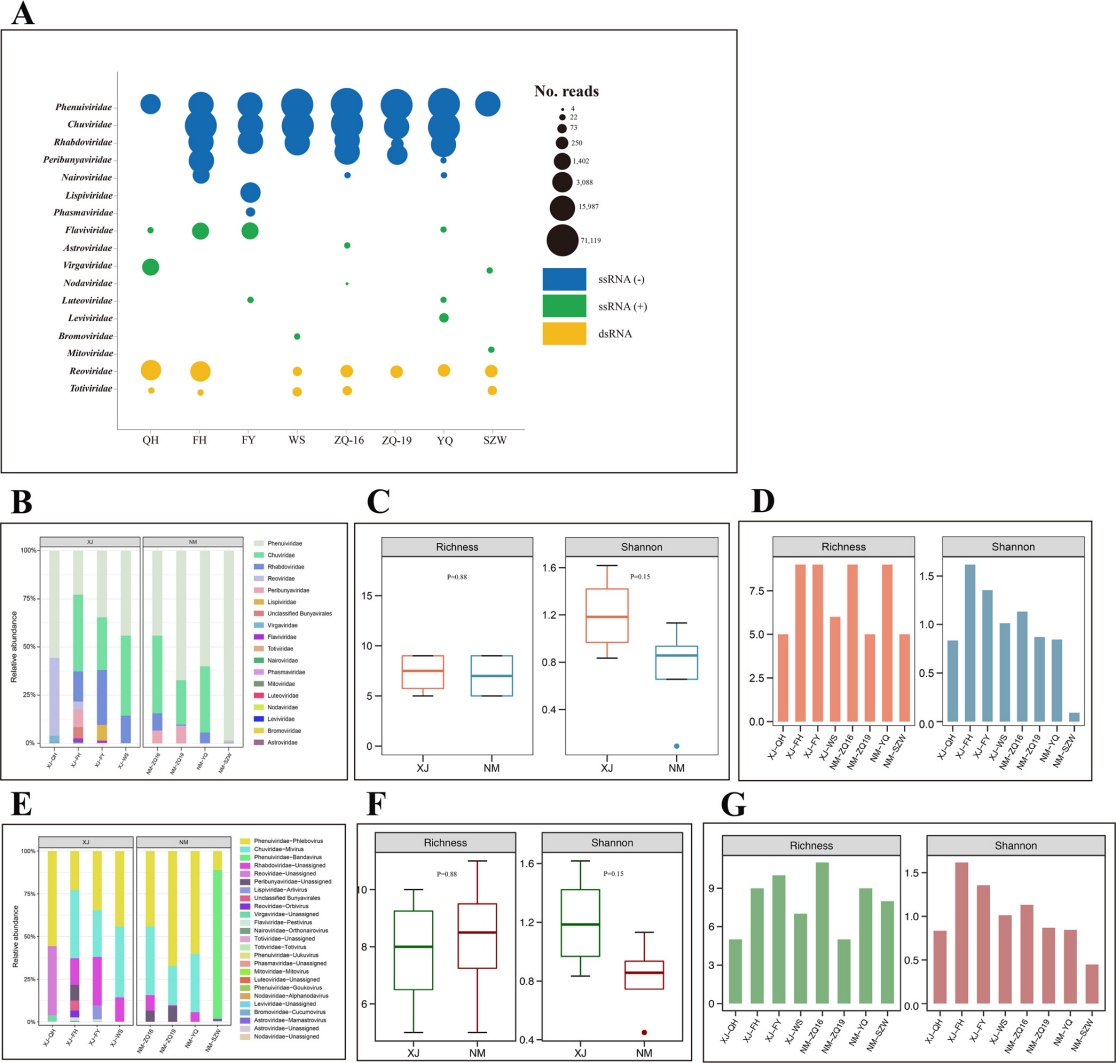
Taxonomic analyses of RNA viral reads at the family or genus level. Viral types or viral families are shown with corresponding colors (see color legend). (A) Bubble plot showing the read abundances of different viral families in the tick virome. Read numbers were log_2_ transformed and are represented by the smallest circle. Abbreviations in the pie charts represent tick collection sites. (B to G) Alpha diversity of viral families or viral genera in the tick libraries, including the abundance and diversity of viral families (B) and genera in the tick libraries (E), the alpha diversity of viral families and genera in Xinjiang (C) and Inner Mongolia (E), and the alpha diversity metrics of viral families (D) and genera (G) for each tick library.

After alignment by BLAST, the viral reads were finally annotated to 17 families. Viruses in the families *Phenuiviridae*, *Chuviridae*, *Rhabdoviridae*, *Peribunyaviridae*, *Nairoviridae*, *Lispiviridae*, and *Phasmaviridae* had single-stranded negative-sense RNA [(−) ssRNA] genomes; viruses in the families *Flaviviridae*, *Astroviridae*, *Virgaviridae*, *Nodaviridae*, *Luteoviridae*, *Leviviridae*, *Bromoviridae*, and *Mitoviridae* had single-stranded positive-sense RNA [(+) ssRNA] genomes; and viruses in the families *Reoviridae* and *Totiviridae* had double-stranded RNA (dsRNA) genomes (Fig. 2A). It was apparent that the abundance levels of viruses among the libraries varied significantly. The highest abundance was observed in the FH tick library, whereas the lowest abundance was observed in the SZW tick library (Fig. 2A). (The tick library abbreviations are as follows: FH, Fuhai; FY, Fuyun; QH, Qinghe; WS, Wusu; ZQ, Alxa Left Banner; YQ, Alxa Right Banner; SZW, Siziwang Banner.)

Alpha diversity (the diversity within each tick library) was highest in FY and FH at both the viral family and genus levels and was lowest in SZW (Fig. 2B, D, E and G). Interestingly, there was no difference in overall virus abundance and diversity among ticks collected from the two autonomous regions (Fig. 2C and F). Although there was variation in viral composition among the tick libraries, members of *Phenuiviridae* were the most abundant in each tick library, comprising 53, 23, 31, 44, 43, 68, 64, and 91% of all the viral reads in these eight libraries, respectively (Fig. 2B). Regarding beta diversity, there were similar viral components in tick libraries from Wusu, Alxa Left Banner, and Alxa Right Banner. They shared many species of viruses, despite their locations being far apart (see Fig. S1 in the supplemental material).

### Discovery and prevalence of tick RNA viruses.

**FIG 3:**
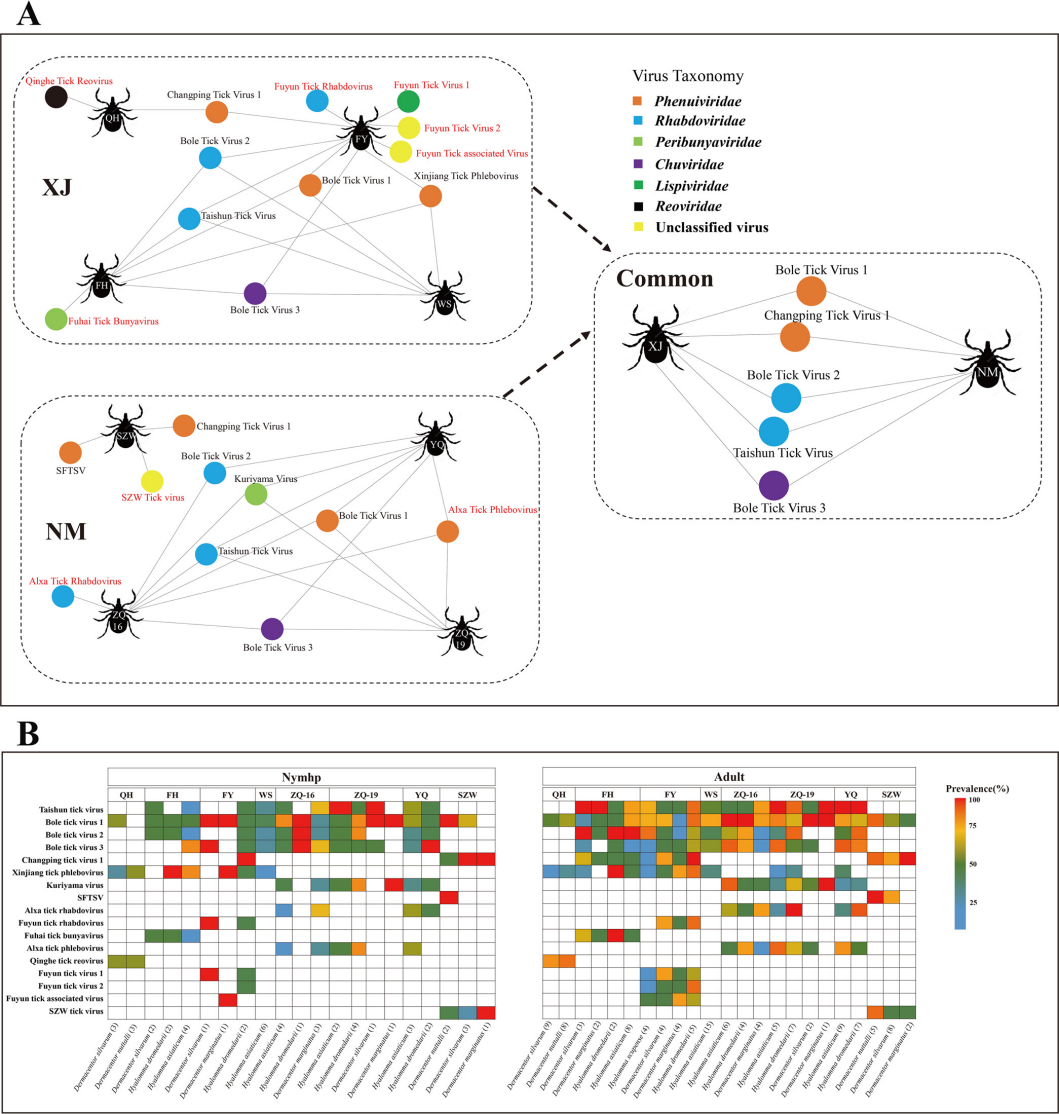
Tick-borne virus discovery and prevalence at the different sampling collection locations. (A) Network plot illustrating biologically relevant virus species for which viral genomes were identified in each tick library. In both panels, each library is represented as a tick icon, with the abbreviation of the sampling location. (See the legend for Fig. 1 for definitions of those abbreviations.) Circles represent viruses, and different colors correspond to virus families. When two databases share a virus type, the network between the two databases is linked. The connector does not represent any variable. (B) Distributions of tick-borne virus prevalence at the different sampling locations. Since the ticks were pooled together during the extraction process, the results are summarized according to the species of ticks, some developmental stages, and collection sites. The positive number of tick pools at each location or in each developmental stage is shown in parentheses.

Seventeen RNA viruses were identified from the eight tick libraries. Among them, 14 viruses belonged to six viral families, including *Phenuiviridae*, *Rhabdoviridae*, *Peribunyaviridae*, *Lispiviridae*, *Chuviridae*, and *Reoviridae* (Fig. 3A) (see below). The remaining three virus species belonged to an unclassified viral family. Notably, 9 of the 17 identified viruses were proposed to be new viral species, as they were highly divergent from any of the previously identified viruses; they were designated Qinghe tick reovirus (QTRV), Fuyun tick rhabdovirus (FTRV), Fuyun tick virus 1 (FTV1), Fuyun tick virus 2 (FTV2), Fuyun tick-associated virus (FTAV), Fuhai tick bunyavirus (FTBV), SZW tick virus (STV), Alxa tick phlebovirus (ATPV), and Alxa tick rhabdovirus (ATRV) (see Table S1). Another seven viruses, including Bole tick virus 1 (BTV1), Changping tick virus 1 (CPTV1), Xinjiang tick phlebovirus (XTPV), Bole tick virus 2 (BTV2), Taishun tick virus (TSTV), Kuriyama virus (KYMV), and Bole tick virus 3 (BTV3), showed close relationships with previously reported tick-associated viruses that have not yet been approved by the International Committee on Taxonomy of Viruses (ICTV) (see Table S1). The other virus, SFTSV, was classified as a known species, as it had a close relationship and identical genomic organization to the ICTV-approved virus (see Table S1). Eight of the 17 virus species shared similarities between the libraries from different locations. In addition, five virus species collected in Xinjiang and Inner Mongolia during the different seasons also shared similarities (Fig. 3A). Interestingly, the viral species discovered in Siziwang Banner did not share similarities with those from the other three sites in the network diagram (Fig. 3A). To understand the prevalence of these viruses in ticks from various regions, the viral species and abundances in all 175 tick pools were evaluated by reverse transcription-PCR (RT-PCR). BTV1 was detected in almost all tick pools (Fig. 3B). In addition to detection in the tick pools from Qinghe and Siziwang Banner, TSTV, BTV2, and BTV3 were detected in ticks from the other six locations (Fig. 3B). BTV2 was not detected in nymph or adult Dermacentor silvarum or Dermacentor marginatus collected from Alxa Left Banner in 2019 (Fig. 3B). The other 13 viruses showed regional endemicity. XTPV was detected in tick pools from four collection sites in Xinjiang but in only adults from ZQ-19 and YQ in Inner Mongolia (Fig. 3B). Remarkably, SFTSV was detected in all tick pools of Dermacentor nuttalli collected in Siziwang Banner but not in tick pools of *Dermacentor marginatus* (Fig. 3B).

### RNA virus diversity and evolution.

**FIG 4:**
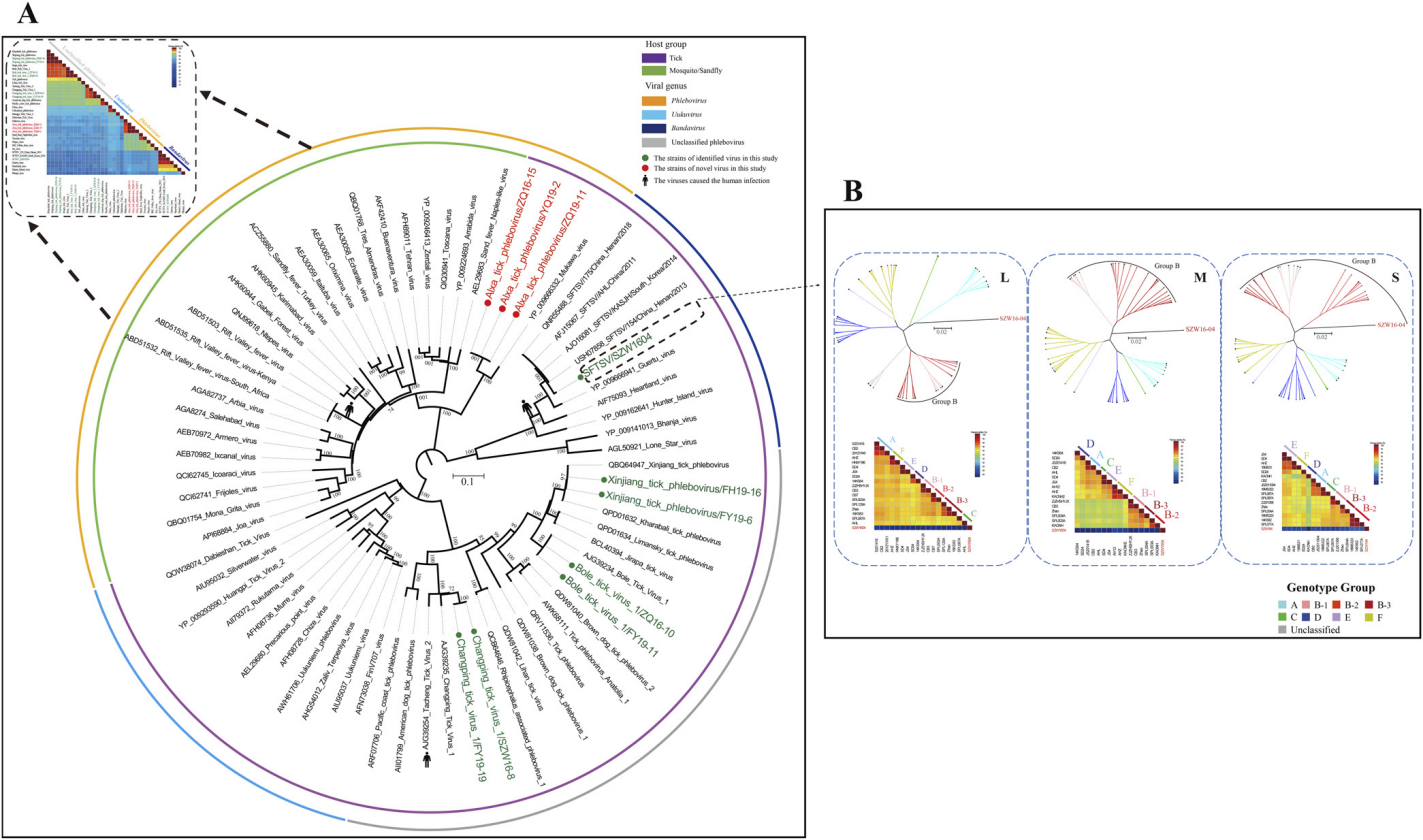
Phylogenetic analysis of *Phenuiviridae*. (A) Phylogenetic tree and pairwise genetic distance heatmap of 3 genera and unclassified viruses in the order *Phenuiviridae*, based on RdRp domain amino acid sequences. Nodes with bootstrap values of ≥70 are indicated. (B) Phylogenetic analysis and pairwise genetic distance heatmap of SFTSV, based on the complete nucleotide coding sequences of the L, M, and S segments. Each scale bar indicates the number of amino acid or nucleotide substitutions per site. The newly identified and previously identified viruses in this study are marked with red and green circles, respectively.

**(i) *Phenuiviridae.*** Viral reads of *Phenuiviridae* were the most abundant in each tick library, and from these reads we assembled five viral genome sequences corresponding to four previously identified viruses, BTV1, CPTV1, XTPV, and SFTSV, and a novel virus tentatively named ATPV. In the phylogenetic tree of the RNA-dependent RNA polymerase (RdRp) domains of *Phenuiviridae*, ATPV and SFTSV belonged to the genera *Phlebovirus* and *Bandavirus*, respectively (Fig. 4A). BTV1, CPTV1, and XTPV were all classified as unclassified phleboviruses (Fig. 4A). Pairwise amino acid comparisons showed that ATPV shared less than 82% sequence similarity to all other genetically related members in *Phenuiviridae*; hence, it represents a novel species (Fig. 4A). ATPV was clustered together with Mukawa virus (GenBank accession number YP_009666332), a virus detected from Ixodes persulcatus sampled in Japan in 2013, and they shared 82.94% identity between their RdRp amino acid sequences, 73.43% identity between their nucleocapsid amino acid sequences, and 62.2% identity between their glycoprotein amino acid sequences (Fig. 4A). Both previously identified viruses clustered together with a formerly reported identical virus species in the phylogenetic tree (Fig. 4A). Moreover, they shared >90% sequence identity with the same viral species discovered earlier (Fig. 4A). Of note, the evolutionary relationship of the SFTSV/SZW1604 strain was different in the nucleotide phylogenic analysis. The strain was in a separate evolutionary clade on the phylogenetic tree of the L and M segments, while this strain was clustered together with group B in the phylogenetic tree of the M segment (Fig. 4B). In addition, the sequence similarity of the three fragments was 88% compared with other SFTSV strains (Fig. 4B).

**FIG 5:**
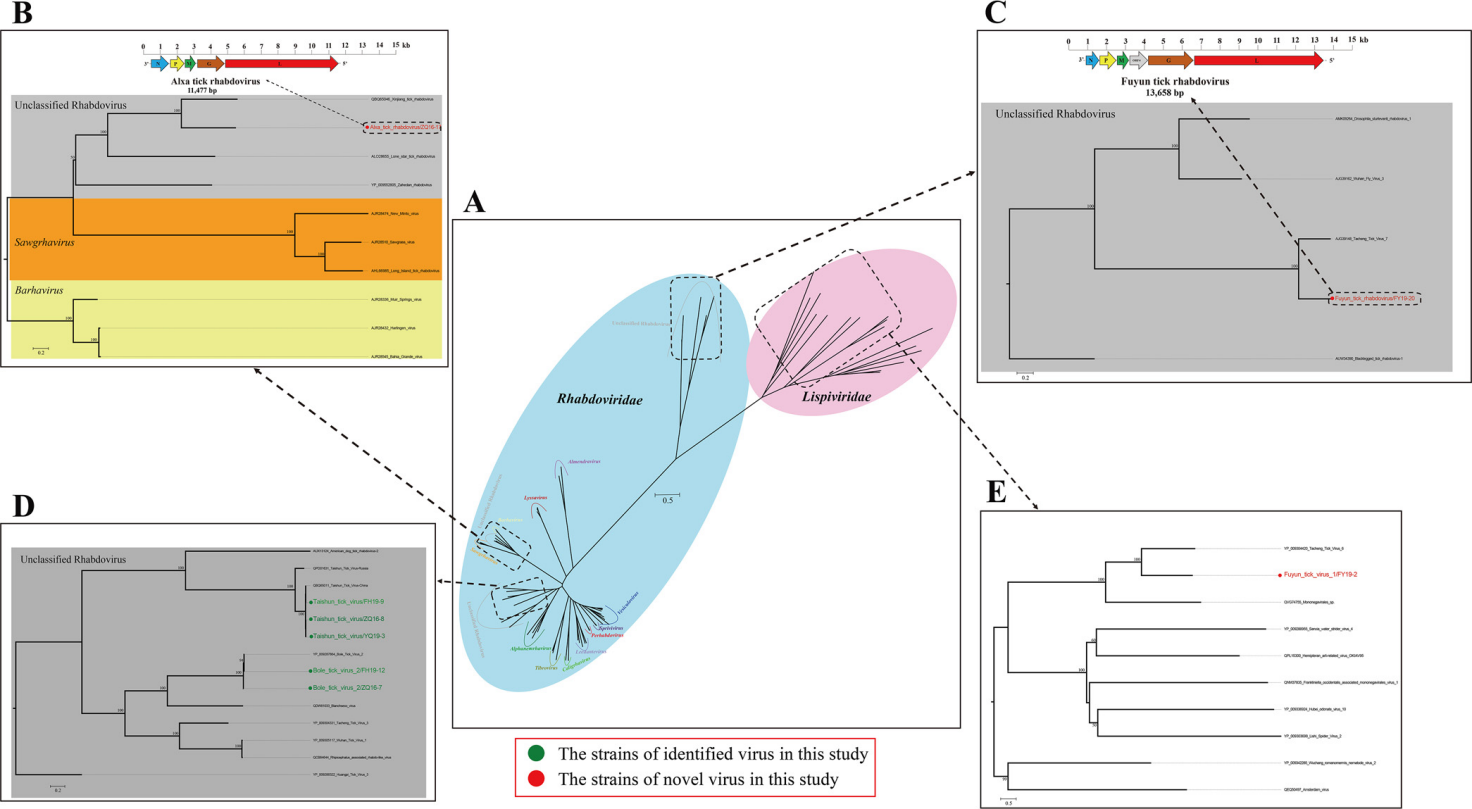
Phylogenetic analysis of *Mononegavirales*. (A) Phylogenetic tree of *Rhabdoviridae* and *Lispiviridae* based on RdRp domain amino acid sequences. (B to D). Phylogenetic trees for novel and previously identified rhabdoviruses in this study and members of closely related viruses in the family *Rhabdoviridae*. (E) Phylogenetic analysis of novel lispivirus in this study and other viruses in the family *Lispiviridae*. Nodes with bootstrap values of ≥70 are indicated. Each scale bar indicates the number of amino acid substitutions per site. The novel and previously identified viruses in this study are marked with red and green circles, respectively.

**(ii) *Rhabdoviridae.*** Viral reads of *Rhabdoviridae* were abundant in the tick libraries, except for the QH and SZW libraries, and we assembled four viral genome sequences corresponding to two previously identified viruses, BTV2 and TSTV, and two new viruses, tentatively named ATRV and FTRV. According to the phylogenic analysis of the RdRp domains of *Mononegavirales*, these four viruses all belonged to unclassified rhabdoviruses (Fig. 5A). ATRV had five open reading frames (ORFs), while FTRV had one more ORF than ATRV (Fig. 5B and C). The virus with the closest evolutionary relationship to ATRV was Xinjiang tick rhabdovirus (GenBank QBQ65046), which was detected in ticks in China (Fig. 5B). Phylogenetic analysis showed that FTRV was clustered together with Tacheng tick virus 7 (Fig. 5C). Similarly, BTV2 and TSTV, previously identified viruses, were clustered together with the same viral species previously reported in the phylogenetic tree (Fig. 5D). Notably, the evolutionary clade of FTRV was closer to that of *Lispiviridae* (Fig. 5A).

**(iii) *Lispiviridae.*** *Lispiviridae*, a relatively new family, was described in 2015 (30). The newly discovered FTV1 fell within family *Lispiviridae*, order *Mononegavirales*; the closest relationship (nucleotide identity, 65.6%) was with Tacheng tick virus 6 (GenBank YP_009304420), which was identified in Argas miniatus in China (Fig. 5E).

**FIG 6:**
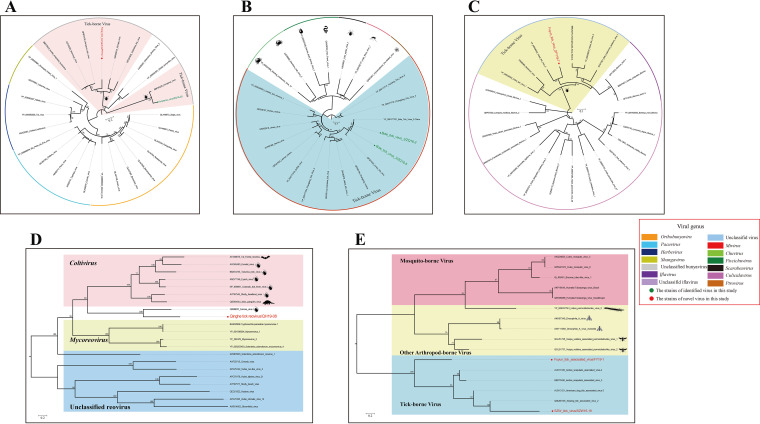
Phylogenetic analysis of other RNA viruses based on RdRp domain amino acid sequences. (A) Phylogenetic tree of 4 genera and unclassified viruses in the order *Peribunyaviridae*. (B) Phylogenetic tree of 6 genera in the order *Chuviridae*. (C and E) Phylogenetic trees for novel unclassified viruses in this study and other viruses with close relationships. (D) Phylogenetic tree for novel viruses in this study and members of the closely related viruses in the family *Reoviridae*. Nodes with bootstrap values of ≥70 are indicated. Each scale bar indicates the number of amino acid substitutions per site. The newly identified and previously identified viruses in this study are marked with red and green circles, respectively.

**(iv) *Peribunyaviridae.*** In the RdRP tree, both peribunyaviruses identified in the current study fell within an unclassified clade, provisionally designated an unclassified peribunyavirus group, but formed a distinct branch (Fig. 6A). KYMV/ZQ16-20 was clustered together with a strain of KYMV that was previously identified in Ixodes persulcatus in Japan (GenBank BBF90225) (Fig. 6A). The closest relationship (nucleotide identity, 69.7%) of FTBV was with Ixodes scapularis bunyavirus (GenBank BBD75425), which was identified in ticks in Japan (Fig. 6A).

**(v) *Chuviridae.*** *Chuviridae*, a relatively new family and species, was discovered in 2015 and classified as a new family by the ICTV in 2018 (30). BTV3 was identified in six tick libraries, excluding the QH and SZW tick libraries. In the phylogenetic relationship of the RdRp domains of *Chuviridae*, BTV3 belonged to the genus *Mivirus* and clustered together with a previous strain of BTV3 that was identified in Hyalomma asiaticum in China (GenBank YP_009177701) (Fig. 6B).

**(vi) *Reoviridae.*** Two reovirus sequences encoding RdRp and VP2 were identified in the QH tick library. These virus sequences were from the newly-reovirus QTRV. Phylogenetic analysis showed that the virus formed an evolutionary branch with the Fennes virus previously found in parasitic penguin ticks in Antarctica (Fig. 6D). Both QTRV and Fennes virus belong to the genus *Coltivirus* in family *Reoviridae* and clustered on the genus’s evolutionary branch. Since only these two genomic fragments of the virus were obtained by sequencing, analysis of the remaining 10 ORF gene fragments was impossible.

### Unclassified viruses.

In the viral reads obtained by total RNA sequencing, abundant viral sequences were annotated as unclassified viruses. Three tick-associated unclassified viral sequences belonging to FTV2, FTAV, and STV were observed in all the tick libraries. Phylogenetically, FTV2 was clustered together with Hubei tick virus 2 (GenBank YP_009336542), a virus discovered from ticks sampled in China in 2013, which also clustered with viruses of the genus *Iflavirus* (Fig. 6C). In the phylogenetic tree of hypothetical proteins of unclassified viruses, FTAV and STV belonged to the evolutionary clade of tick-borne viruses (Fig. 6E). In addition, STV was clustered together with Xinjiang tick-associated virus 2 (GenBank QBQ65105), a virus detected in Dermacentor nuttalli sampled in China in 2015.

## DISCUSSION

As vectors and natural reservoirs of a variety of pathogenic viruses, ticks pose a great threat to public and animal health (31). In recent years, with the development of NGS metagenomics, a variety of tick-borne viruses have been identified (20, 23, 32–34). The discovery of these new TBVs has expanded the spectrum and diversity of TBVs. Here, we examined eight tick viromes of ticks from Xinjiang and Inner Mongolia by metatranscriptomic approaches and revealed diversities in the species and the abundance of harbored viruses in different sampling locations. Although our analyses were limited to eight locations in these two regions, highly diverse and abundant viromes were identified, further demonstrating the capacity of arthropods to tolerate large numbers of viruses (14, 35–38). Indeed, 17 viral species from six known families and an unclassified viral family of RNA viruses were identified in this study. Among these, nine were novel virus species, and the remaining eight were previously identified species, including SFTSV, which is relevant to human health (Fig. 3). Importantly, these novel viruses were likely tick viruses, because they were closely related to previously identified viruses in ticks or other arthropod species. They mostly had high prevalence rates and abundances in the tick libraries; therefore, it is unlikely that they were viruses associated with herbivore livestock (Fig. 4 and 6). In addition, the viral sequences of *Astroviridae* and the genus *Pestivirus* of *Flaviviridae* were detected in five tick libraries (Fig. 2A). These viruses are able to infect ruminants, suggesting that some ticks had ingested host blood before sample collection. It is currently unknown whether ticks are natural hosts of astroviruses and pestiviruses. These two groups of viruses have been detected in multiple mammal species, but only astroviruses have been detected in arthropods (35, 39, 40). Notably, rabbit astroviru*s* was detected in the virome of rabbit-associated ectoparasites, but it was not detected in rabbits (41). This finding indicates that ectoparasites might be involved in vertebrate-associated virus transmission, but the viruses are not carried by the ectoparasites themselves. The presence of arthropod-specific viruses in ticks might impact the replication and transmission of arboviruses that also infect vertebrates, which has been demonstrated both *in vitro* and *in vivo* (42–45). Viruses in the family *Leviviridae* were also identified in this study. This may not be surprising, since fungi are natural hosts of viruses in families such as *Totiviridae* and *Mitoviridae*, and ticks can be contaminated by environmental fungi, bacteria, and other flora (46–48).

Metatranscriptomic analysis can reveal the diversity and abundance of total viromes and is commonly used to explore viromes among different host species, geographical distributions, seasonal environments, and sampling years (34, 49–52). Interestingly, in this study, ticks from different sampling locations carried the same viruses, despite the great distance between the samples (Fig. 3A). BTV1, BTV2, BTV3, CPTV1, and TSTV were all observed in the six sampling locations, revealing a potential connection between these tick populations. Whether this was due to similarities in tick species and geographical environments requires further investigation. In addition, Xinjiang is adjacent to northwestern Inner Mongolia, which provides suitable conditions for the migration of natural hosts of ticks, such as wild animals (25). Another possible reason for the similar virus species is that the two regions' common viruses originated in the same hosts. Later, ticks carrying these viruses were transported to other locations through the livestock trade or human activity. The global circulation of CCHFV is an example of this (53). In addition, the climatic environments of the eight sampling points are not identical. Season and year of tick collection may also impact the results. For example, numerous significant differences were detected between tick samples collected from Alxa Left Banner in 2016 and in 2019. There are many reasons for this phenomenon. First, most tick-borne viruses are not transmitted vertically, and ticks feed on multiple hosts during their life time; second, differences in environments and tick species may alter the diversity and abundance of TBVs (33). Ticks sampled in Xinjiang Uyghur Autonomous Region and Inner Mongolia Autonomous Region had similar alpha diversity values at the virus family and genus levels. Five species of viruses were detected in Xinjiang and Inner Mongolia (Fig. 3B). In addition, the novel tick-borne viruses found in this study exhibited regional diversity. Our previous study showed that Alxa League (including Alxa Left Banner and Alxa Right Banner) was an area of CCHFV endemicity (54, 55). However, in the present study, we did not detect CCHFV in this region. This phenomenon may be due to sampling limitations and because tick dispersal to different regions is reliant on hosts. Interestingly, SFTSV was detected in both adult and nymph *Dermacentor nuttalli* pools, and it was found in adult *Dermacentor silvarum* but not *Dermacentor marginatus* (Fig. 3B). This indicates that *Dermacentor nuttalli* may be a reservoir of SFTSV; whether *Dermacentor silvarum* and *Dermacentor marginatus* are also reservoirs for SFTSV should be further studied in a larger sample size.

Phylogenetic analysis showed comprehensive evolutionary relationships between these tick-borne RNA viruses. All the viruses in this study clustered on the tick-borne virus clade in the phylogenetic tree and had evolutionary characteristics similar to tick-borne viruses reported in previous studies (34, 56). All the identified viruses showed characteristics closely related to ticks or arthropods, indicating that they might be transmitted between ticks, without host spillover (Fig. 4 and 6). In addition, SFTSV, identified in this study, has a pathogenic relationship with humans. It is a known highly pathogenic tick-borne virus and has been detected and reported in many regions and countries. It is therefore not surprising that it was detected in ticks in Inner Mongolia (9). Of interest, the SZW1604 strain identified in this study has a unique evolutionary relationship with SFTSV. Because SFTSV can spread between different regions through bird migration, the viral transmission range could be expanded (57). Moreover, rearrangement and recombination occur frequently among SFTSV strains and are usually not limited to viruses in specific regions, facilitating viral evolution (58, 59). Notably, QTRV was phylogenetically clustered with the genus *Coltivirus*, which includes the pathogenic tick-borne virus Colorado tick fever virus and a number of tick-associated viruses identified in African bats (34, 42, 56–61). In this regard, QTRV is in a relatively basic position in the genus *Coltivirus* and its specific evolution. It is currently uncertain whether ticks are the only host of this virus or whether vertebrates also serve as hosts, since other reoviruses in this genus are also suspected to infect vertebrates, including rodents (34). Notably, the virus genome lacks segments 3 to 12, and it has been proposed that these missing segments were not identified probably due to low similarities between QTRV and known reoviruses (61–63).

This study has several limitations. First, we performed mixed sequencing by pooling ticks from different sampling locations, which did not reflect differences in the diversity and abundance of viromes according to tick species. Second, the frequency of tick sampling was low, and the quantities of samples were small, which may not fully reflect the relationships between tick viromes in various regions. Third, data on some potentially important parameters, such as temperature, humidity, and anthropogenic activities, were not collected, limiting our capability to reveal multiple factors that influence virome structures. Finally, we focused on only the genetic characteristics of novel viruses in this study. The potential pathogenicity of the novel viruses identified, such as QTRV and ATPV, in herbivore livestock, humans, and other mammals remains unknown.

In conclusion, in this study we analyzed the diversity of tick viruses in Xinjiang and Inner Mongolia and revealed the phylogenetic relationships of some RNA viruses. These viruses showed a complex evolutionary history and potential pathogenicity. Therefore, it is necessary to further investigate tick-borne viruses in the Xinjiang and Inner Mongolia regions. In addition, as the pathogenicity of these tick-borne viruses remains unknown, future studies should focus on evaluating the transmission ability and pathogenicity of these viruses; such studies will provide valuable information for the creation of strategies for the prevention and control of infectious diseases caused by tick-borne viruses.

## MATERIALS AND METHODS

### Sample collection.

In 2016 and 2019, parasitic ticks were collected from domestic animals (camels and sheep) in four regions of Xinjiang Uygur Autonomous Region (Wusu, Qinghe, Fuyun, and Fuhai) and four regions of Inner Mongolia (Siziwang Banner, Alxa Left Banner, Right Banner, and the junction of the left and right banners) (Fig. 1). The samples were transported to the laboratory on dry ice and frozen at −80°C. The ticks were initially identified to the species level by morphological characteristics, and then the species was confirmed based on PCR amplification and sequencing of the cytochrome *c* oxidase subunit 1 gene (64–66). All the ticks were grouped into 175 pools by species and sampling location (*n* = 12 to 15 ticks per pool). Owners of animals were informed about the aims and process of this study. All the owners and farmers agreed to the collection of ticks from their camels and sheep.

### Nucleic acid extraction, RNA library construction, and sequencing.

Before RNA extraction, ticks were homogenized in a frozen grinder (Jingxin, Shanghai, China). RNA was extracted using a viral RNA minikit (Qiagen) according to the manufacturer's instructions. The quality and concentration of extracted RNA were evaluated with an Agilent 2100 Bioanalyzer. Host rRNA was removed using the Hieff NGS MaxUp rRNA depletion kit (Yeasen, Shanghai, China) prior to constructing the total RNA library using the TruSeq total RNA library preparation scheme (Illumina) for sequencing analysis. RNA sequencing was performed on the Illumina HiSeq XTen platform at Shaanxi Xuanchen Biotechnology Co., Ltd. (Shaanxi, China).

### Acquisition of the viral genome.

The obtained sequencing reads were filtered and trimmed using Trimmomatic for quality control (67). The resulting reads (non-rRNA) were then *de novo* assembled using the Trinity program with default settings (68). Next, BLASTn and Diamond BLASTx were used to compare all assembled contigs with the whole nonredundant nucleotide (NT) and protein (NR) databases, respectively. The E-value threshold was set to 1 × 10^−5^ to remove false positives (69, 70). Taxonomic classifications of BLASTn and Diamond BLASTx results were parsed using MEGAN to run the last common ancestor (LCA) assignment algorithm according to default parameters (71). For viruses with large differences in sequences from known viruses, BLASTx was used to verify whether the sequences were from a novel virus. The SeqMan program implemented in the Lasergene software package v5.0 (DNAstar, Madison, WI, USA) was used to merge contigs with unassembled overlaps (72). To eliminate misassembly, all of the rRNA reads were removed with Bowtie2 and inspected using Geneious v11.1.2 (73, 74). For newly obtained virus sequences, potential ORFs were predicted using ORF Finder (75).

### Phylogenetic analysis.

The obtained data set of complete and partial viral sequences was compared with the reference sequences in the NCBI database using MAFFT (76). RdRp (RNA-dependent RNA polymerase), nucleoprotein, and other protein sequences were used for phylogenetic analysis. Maximum-likelihood (ML) phylogenies were inferred using IQ-TREE under the best-fit substitution model for 10,000 ultrafast bootstraps (77, 78). The best model of trees was calculated by Modelfinder (79). The phylogenetic trees were edited and visualized with FigTree v.1.4.4 (http://tree.bio.ed.ac.uk/software/figtree). Pairwise identities between the RdRp protein sequences of *Phenuiviridae* and the genomic sequences of SFTSV were calculated using SDT v1.2 (80).

### Analysis of diversity and abundance of viruses.

The primarily annotated viral sequences were verified by BLASTx and further annotated to specific family, genus, and other classification groups for each read in each sequencing library. Unclassified viral reads were separately annotated and were not included in the diversity analysis. To comprehensively evaluate the alpha diversity of viruses in these libraries, the richness of each RNA sequencing library was characterized by viral family and viral genus, and the diversity and abundance of sequence groups were characterized by Shannon and richness indexes. Both the tick virome richness and Shannon alpha diversity were calculated for each library at the virus family and genus levels using the Rhea package and compared to detect differences between tick libraries using the Kruskal-Wallis rank-sum test (81). Analyses were performed using R v4.0.2 integrated into RStudio v1.3.1093 and plotted using the ggplot2 package. For the beta diversity analysis, the Bray-Curtis similarity matrix calculation was first performed. Then, the vegan and ggplot2 packages were used for nonmetric multidimensional analysis to characterize the structural distribution of virus information at the family and genus levels in each RNA sequencing library.

### Data availability.

All sequence reads generated in this project are available in the NCBI Short Read Archive under BioProject PRJNA871396. All viral genome sequences have been submitted to the GenBank database under accession numbers OP312991 to OP313026 (see Table S1 in the supplemental material).

## References

[1] Yu Z, Wang H, Wang T, Sun W, Yang X, Liu J. 2015. Tick-borne pathogens and the vector potential of ticks in China. Parasit Vectors 8:24. doi:10.1186/s13071-014-0628-x. 25586007 PMC4300027

[2] Shi J, Hu Z, Deng F, Shen S. 2018. Tick-borne viruses. Virol Sin 33:21–43. doi:10.1007/s12250-018-0019-0. 29536246 PMC5866268

[3] Kazimirova M, Thangamani S, Bartikova P, Hermance M, Holikova V, Stibraniova I, Nuttall PA. 2017. Tick-borne viruses and biological processes at the tick-host-virus interface. Front Cell Infect Microbiol 7:339. doi:10.3389/fcimb.2017.00339. 28798904 PMC5526847

[4] Yoshii K, Song JY, Park SB, Yang J, Schmitt HJ. 2017. Tick-borne encephalitis in Japan, Republic of Korea and China. Emerg Microbes Infect 6:e82. https://www.tandfonline.com/doi/full/10.1038/emi.2017.69. 28928417 10.1038/emi.2017.69 PMC5625319

[5] Guo R, Shen S, Zhang Y, Shi J, Su Z, Liu D, Liu J, Yang J, Wang Q, Hu Z, Zhang Y, Deng F. 2017. A new strain of Crimean-Congo hemorrhagic fever virus isolated from Xinjiang, China. Virol Sin 32:80–88. doi:10.1007/s12250-016-3936-9. 28251517 PMC6598972

[6] Zhang Y, Shen S, Fang Y, Liu J, Su Z, Liang J, Zhang Z, Wu Q, Wang C, Abudurexiti A, Hu Z, Zhang Y, Deng F. 2018. Isolation, characterization, and phylogenetic analysis of two new Crimean-Congo hemorrhagic fever virus strains from the northern region of Xinjiang Province, China. Virol Sin 33:74–86. doi:10.1007/s12250-018-0020-7. 29520745 PMC6178084

[7] Liu Q, He B, Huang S-Y, Wei F, Zhu X-Q. 2014. Severe fever with thrombocytopenia syndrome, an emerging tick-borne zoonosis. Lancet Infect Dis 14:763–772. doi:10.1016/S1473-3099(14)70718-2. 24837566

[8] Yu XJ, Liang MF, Zhang SY, Liu Y, Li JD, Sun YL, Zhang L, Zhang QF, Popov VL, Li C, Qu J, Li Q, Zhang YP, Hai R, Wu W, Wang Q, Zhan FX, Wang XJ, Kan B, Wang SW, Wan KL, Jing HQ, Lu JX, Yin WW, Zhou H, Guan XH, Liu JF, Bi ZQ, Liu GH, Ren J, Wang H, Zhao Z, Song JD, He JR, Wan T, Zhang JS, Fu XP, Sun LN, Dong XP, Feng ZJ, Yang WZ, Hong T, Zhang Y, Walker DH, Wang Y, Li DX. 2011. Fever with thrombocytopenia associated with a novel bunyavirus in China. N Engl J Med 364:1523–1532. doi:10.1056/NEJMoa1010095. 21410387 PMC3113718

[9] Li J, Li S, Yang L, Cao P, Lu J. 2021. Severe fever with thrombocytopenia syndrome virus: a highly lethal bunyavirus. Crit Rev Microbiol 47:112–125. doi:10.1080/1040841X.2020.1847037. 33245676

[10] Tran XC, Yun Y, Van An L, Kim SH, Thao NTP, Man PKC, Yoo JR, Heo ST, Cho NH, Lee KH. 2019. Endemic severe fever with thrombocytopenia syndrome, Vietnam. Emerg Infect Dis 25:1029–1031. doi:10.3201/eid2505.181463. 31002059 PMC6478219

[11] Ma J, Chen H, Gao X, Xiao J, Wang H. 2020. African swine fever emerging in China: distribution characteristics and high-risk areas. Prev Vet Med 175:104861. doi:10.1016/j.prevetmed.2019.104861. 31810030

[12] Mighell E, Ward MP. 2021. African Swine fever spread across Asia, 2018–2019. Transbound Emerg Dis 68:2722–2732. doi:10.1111/tbed.14039. 33599077

[13] Li CX, Shi M, Tian JH, Lin XD, Kang YJ, Chen LJ, Qin XC, Xu J, Holmes EC, Zhang YZ. 2015. Unprecedented genomic diversity of RNA viruses in arthropods reveals the ancestry of negative-sense RNA viruses. Elife 4 doi:10.7554/eLife.05378. PMC4384744 25633976

[14] Shi M, Lin XD, Tian JH, Chen LJ, Chen X, Li CX, Qin XC, Li J, Cao JP, Eden JS, Buchmann J, Wang W, Xu J, Holmes EC, Zhang YZ. 2016. Redefining the invertebrate RNA virosphere. Nature 540:539–543. doi:10.1038/nature20167. 27880757

[15] Dutilh BE, Reyes A, Hall RJ, Whiteson KL. 2017. Virus discovery by metagenomics: the (im)possibilities. Front Microbiol 8:1710. doi:10.3389/fmicb.2017.01710. 28943867 PMC5596103

[16] Nooij S, Schmitz D, Vennema H, Kroneman A, Koopmans MPG. 2018. Overview of virus metagenomic classification methods and their biological applications. Front Microbiol 9:749. doi:10.3389/fmicb.2018.00749. 29740407 PMC5924777

[17] Coffey LL, Page BL, Greninger AL, Herring BL, Russell RC, Doggett SL, Haniotis J, Wang C, Deng X, Delwart EL. 2014. Enhanced arbovirus surveillance with deep sequencing: Identification of novel rhabdoviruses and bunyaviruses in Australian mosquitoes. Virology 448:146–158. doi:10.1016/j.virol.2013.09.026. 24314645 PMC3870893

[18] Remnant EJ, Shi M, Buchmann G, Blacquiere T, Holmes EC, Beekman M, Ashe A. 2017. A diverse range of novel RNA viruses in geographically distinct honey bee populations. J Virol 91. doi:10.1128/JVI.00158-17. PMC5533899 28515299

[19] Modha S, Hughes J, Bianco G, Ferguson HM, Helm B, Tong L, Wilkie GS, Kohl A, Schnettler E. 2019. Metaviromics reveals unknown viral diversity in the biting midge Culicoides impunctatus. Viruses 11:865. doi:10.3390/v11090865. PMC6784199 31533247

[20] Pettersson JH, Shi M, Bohlin J, Eldholm V, Brynildsrud OB, Paulsen KM, Andreassen A, Holmes EC. 2017. Characterizing the virome of Ixodes ricinus ticks from northern Europe. Sci Rep 7:10870. doi:10.1038/s41598-017-11439-y. 28883464 PMC5589870

[21] Gomez GF, Isaza JP, Segura JA, Alzate JF, Gutierrez LA. 2020. Metatranscriptomic virome assessment of Rhipicephalus microplus from Colombia. Ticks Tick Borne Dis 11:101426. doi:10.1016/j.ttbdis.2020.101426. 32473925

[22] Sameroff S, Tokarz R, Jain K, Oleynik A, Carrington CVF, Lipkin WI, Oura CAL. 2021. Novel quaranjavirus and other viral sequences identified from ticks parasitizing hunted wildlife in Trinidad and Tobago. Ticks Tick Borne Dis 12:101730. doi:10.1016/j.ttbdis.2021.101730. 33957484

[23] Shi J, Shen S, Wu H, Zhang Y, Deng F. 2021. Metagenomic profiling of viruses associated with Rhipicephalus microplus ticks in Yunnan Province, China. Virol Sin 36:623–635. doi:10.1007/s12250-020-00319-x. 33400089 PMC8379324

[24] Xu L, Guo M, Hu B, Zhou H, Yang W, Hui L, Huang R, Zhan J, Shi W, Wu Y. 2021. Tick virome diversity in Hubei Province, China, and the influence of host ecology. Virus Evol 7:veab089. doi:10.1093/ve/veab089. 34804590 PMC8599308

[25] Zhang YK, Zhang XY, Liu JZ. 2019. Ticks (Acari: Ixodoidea) in China: geographical distribution, host diversity, and specificity. Arch Insect Biochem Physiol 102:e21544. doi:10.1002/arch.21544. 30859631 PMC6850514

[26] Wang ZD, Wang B, Wei F, Han SZ, Zhang L, Yang ZT, Yan Y, Lv XL, Li L, Wang SC, Song MX, Zhang HJ, Huang SJ, Chen J, Huang FQ, Li S, Liu HH, Hong J, Jin YL, Wang W, Zhou JY, Liu Q. 2019. A new segmented virus associated with human febrile illness in China. N Engl J Med 380:2116–2125. doi:10.1056/NEJMoa1805068. 31141633

[27] Liu X, Zhang X, Wang Z, Dong Z, Xie S, Jiang M, Song R, Ma J, Chen S, Chen K, Zhang H, Si X, Li C, Jin N, Wang Y, Liu Q. 2020. A tentative tamdy Orthonairovirus related to febrile illness in Northwestern China. Clin Infect Dis 70:2155–2160. doi:10.1093/cid/ciz602. 31260510

[28] Dong Z, Yang M, Wang Z, Zhao S, Xie S, Yang Y, Liu G, Zhao S, Xie J, Liu Q, Wang Y. 2021. Human Tacheng tick virus 2 infection, China, 2019. Emerg Infect Dis 27:594–598. doi:10.3201/eid2702.191486. 33496245 PMC7853585

[29] Ma J, Lv XL, Zhang X, Han SZ, Wang ZD, Li L, Sun HT, Ma LX, Cheng ZL, Shao JW, Chen C, Zhao YH, Sui L, Liu LN, Qian J, Wang W, Liu Q. 2021. Identification of a new orthonairovirus associated with human febrile illness in China. Nat Med 27:434–439. doi:10.1038/s41591-020-01228-y. 33603240

[30] Kuhn JH, Adkins S, Agwanda BR, Al Kubrusli R, Alkhovsky SV, Amarasinghe GK, Avsic-Zupanc T, Ayllon MA, Bahl J, Balkema-Buschmann A, Ballinger MJ, Basler CF, Bavari S, Beer M, Bejerman N, Bennett AJ, Bente DA, Bergeron E, Bird BH, Blair CD, Blasdell KR, Blystad DR, Bojko J, Borth WB, Bradfute S, Breyta R, Briese T, Brown PA, Brown JK, Buchholz UJ, Buchmeier MJ, Bukreyev A, Burt F, Buttner C, Calisher CH, Cao M, Casas I, Chandran K, Charrel RN, Cheng Q, Chiaki Y, Chiapello M, Choi IR, Ciuffo M, Clegg JCS, Crozier I, Dal Bo E, de la Torre JC, de Lamballerie X, de Swart RL, et al. 2021. Taxonomic update of phylum Negarnaviricota (Riboviria: Orthornavirae), including the large orders Bunyavirales and Mononegavirales. Arch Virol 166:3513–3566. doi:10.1007/s00705-021-05143-6. 34463877 PMC8627462

[31] Vandegrift KJ, Kapoor A. 2019. The ecology of new constituents of the tick virome and their relevance to public health. Viruses 11:529. doi:10.3390/v11060529. PMC6630940 31181599

[32] Souza WM, Fumagalli MJ, Torres Carrasco AO, Romeiro MF, Modha S, Seki MC, Gheller JM, Daffre S, Nunes MRT, Murcia PR, Acrani GO, Figueiredo LTM. 2018. Viral diversity of Rhipicephalus microplus parasitizing cattle in southern Brazil. Sci Rep 8:16315. doi:10.1038/s41598-018-34630-1. 30397237 PMC6218518

[33] Pettersson JH, Ellstrom P, Ling J, Nilsson I, Bergstrom S, Gonzalez-Acuna D, Olsen B, Holmes EC. 2020. Circumpolar diversification of the Ixodes uriae tick virome. PLoS Pathog 16:e1008759. doi:10.1371/journal.ppat.1008759. 32745135 PMC7425989

[34] Wille M, Harvey E, Shi M, Gonzalez-Acuna D, Holmes EC, Hurt AC. 2020. Sustained RNA virome diversity in Antarctic penguins and their ticks. ISME J 14:1768–1782. doi:10.1038/s41396-020-0643-1. 32286545 PMC7305176

[35] Wu H, Pang R, Cheng T, Xue L, Zeng H, Lei T, Chen M, Wu S, Ding Y, Zhang J, Shi M, Wu Q. 2020. Abundant and diverse RNA viruses in insects revealed by RNA-Seq analysis: ecological and evolutionary implications. mSystems 5:e00039-20. doi:10.1128/mSystems.00039-20. 32636338 PMC7343303

[36] Feng Y, Gou QY, Yang WH, Wu WC, Wang J, Holmes EC, Liang G, Shi M. 2022. A time-series meta-transcriptomic analysis reveals the seasonal, host, and gender structure of mosquito viromes. Virus Evol 8:veac006. doi:10.1093/ve/veac006. 35242359 PMC8887699

[37] Guo L, Lu X, Liu X, Li P, Wu J, Xing F, Peng H, Xiao X, Shi M, Liu Z, Li XD, Guo D. 2021. Meta-transcriptomic analysis reveals the virome and viral genomic evolution of medically important mites. J Virol 95. doi:10.1128/JVI.01686-20. PMC8092686 33208452

[38] Xu Z, Feng Y, Chen X, Shi M, Fu S, Yang W, Liu WJ, Gao GF, Liang G. 2022. Virome of bat-infesting arthropods: highly divergent viruses in different vectors. J Virol 96:e0146421. doi:10.1128/JVI.01464-21. 34586860 PMC8865543

[39] Wu Z, Han Y, Liu B, Li H, Zhu G, Latinne A, Dong J, Sun L, Su H, Liu L, Du J, Zhou S, Chen M, Kritiyakan A, Jittapalapong S, Chaisiri K, Buchy P, Duong V, Yang J, Jiang J, Xu X, Zhou H, Yang F, Irwin DM, Morand S, Daszak P, Wang J, Jin Q. 2021. Decoding the RNA viromes in rodent lungs provides new insight into the origin and evolutionary patterns of rodent-borne pathogens in Mainland Southeast Asia. Microbiome 9:18. doi:10.1186/s40168-020-00965-z. 33478588 PMC7818139

[40] He WT, Hou X, Zhao J, Sun J, He H, Si W, Wang J, Jiang Z, Yan Z, Xing G, Lu M, Suchard MA, Ji X, Gong W, He B, Li J, Lemey P, Guo D, Tu C, Holmes EC, Shi M, Su S. 2022. Virome characterization of game animals in China reveals a spectrum of emerging pathogens. Cell 185:1117–1129.e8. doi:10.1016/j.cell.2022.02.014. 35298912 PMC9942426

[41] Mahar JE, Shi M, Hall RN, Strive T, Holmes EC. 2020. Comparative analysis of RNA virome composition in rabbits and associated ectoparasites. J Virol 94. doi:10.1128/JVI.02119-19. PMC7269439 32188733

[42] Fujita R, Ejiri H, Lim CK, Noda S, Yamauchi T, Watanabe M, Kobayashi D, Takayama-Ito M, Murota K, Posadas-Herrera G, Minami S, Kuwata R, Yamaguchi Y, Horiya M, Katayama Y, Shimoda H, Saijo M, Maeda K, Mizutani T, Isawa H, Sawabe K. 2017. Isolation and characterization of Tarumizu tick virus: a new coltivirus from Haemaphysalis flava ticks in Japan. Virus Res 242:131–140. doi:10.1016/j.virusres.2017.09.017. 28964878

[43] Ejiri H, Lim CK, Isawa H, Fujita R, Murota K, Sato T, Kobayashi D, Kan M, Hattori M, Kimura T, Yamaguchi Y, Takayama-Ito M, Horiya M, Posadas-Herrera G, Minami S, Kuwata R, Shimoda H, Maeda K, Katayama Y, Mizutani T, Saijo M, Kaku K, Shinomiya H, Sawabe K. 2018. Characterization of a novel thogotovirus isolated from Amblyomma testudinarium ticks in Ehime, Japan: a significant phylogenetic relationship to Bourbon virus. Virus Res 249:57–65. doi:10.1016/j.virusres.2018.03.004. 29548745

[44] Shen S, Duan X, Wang B, Zhu L, Zhang Y, Zhang J, Wang J, Luo T, Kou C, Liu D, Lv C, Zhang L, Chang C, Su Z, Tang S, Qiao J, Moming A, Wang C, Abudurexiti A, Wang H, Hu Z, Zhang Y, Sun S, Deng F. 2018. A novel tick-borne phlebovirus, closely related to severe fever with thrombocytopenia syndrome virus and Heartland virus, is a potential pathogen. Emerg Microbes Infect 7:95. doi:10.1038/s41426-018-0093-2. 29802259 PMC5970217

[45] Matsuno K, Kajihara M, Nakao R, Nao N, Mori-Kajihara A, Muramatsu M, Qiu Y, Torii S, Igarashi M, Kasajima N, Mizuma K, Yoshii K, Sawa H, Sugimoto C, Takada A, Ebihara H. 2018. The unique phylogenetic position of a novel tick-borne Phlebovirus ensures an Ixodid origin of the genus Phlebovirus. mSphere 3. doi:10.1128/mSphere.00239-18. PMC6001614 29898985

[46] Hayes SF, Burgdorfer W, Barbour AG. 1983. Bacteriophage in the Ixodes dammini spirochete, etiological agent of Lyme disease. J Bacteriol 154:1436–1439. doi:10.1128/jb.154.3.1436-1439.1983. 6853449 PMC217620

[47] Qiu Y, Abe T, Nakao R, Satoh K, Sugimoto C. 2019. Viral population analysis of the taiga tick, Ixodes persulcatus, by using batch learning self-organizing maps and BLAST search. J Vet Med Sci 81:401–410. doi:10.1292/jvms.18-0483. 30674747 PMC6451905

[48] Rochlin I, Toledo A. 2020. Emerging tick-borne pathogens of public health importance: a mini-review. J Med Microbiol 69:781–791. doi:10.1099/jmm.0.001206. 32478654 PMC7451033

[49] Atoni E, Wang Y, Karungu S, Waruhiu C, Zohaib A, Obanda V, Agwanda B, Mutua M, Xia H, Yuan Z. 2018. Metagenomic virome analysis of Culex mosquitoes from Kenya and China. Viruses 10:30. doi:10.3390/v10010030. PMC5795443 29329230

[50] Wille M, Eden JS, Shi M, Klaassen M, Hurt AC, Holmes EC. 2018. Virus-virus interactions and host ecology are associated with RNA virome structure in wild birds. Mol Ecol 27:5263–5278. doi:10.1111/mec.14918. 30375075 PMC6312746

[51] Wille M, Shi M, Klaassen M, Hurt AC, Holmes EC. 2019. Virome heterogeneity and connectivity in waterfowl and shorebird communities. ISME J 13:2603–2616. doi:10.1038/s41396-019-0458-0. 31239538 PMC6775988

[52] Sidharthan VK, Sevanthi AM, Venkadesan S, Diksha D, Baranwal VK. 2022. Seasonal dynamics in leaf viromes of grapevines depicting leafroll syndrome under tropical condition. Trop Plant Pathol doi:10.1007/s40858-022-00524-x.

[53] Zhou Z, Deng F, Han N, Wang H, Sun S, Zhang Y, Hu Z, Rayner S. 2013. Reassortment and migration analysis of Crimean-Congo haemorrhagic fever virus. J Gen Virol 94:2536–2548. doi:10.1099/vir.0.056374-0. 23939975

[54] Li Y, Yan C, Liu D, He B, Tu C. 2020. Seroepidemiological Investigation of Crimean-Congo hemorrhagic fever virus in sheep and camels of Inner Mongolia of China. Vector Borne Zoonotic Dis 20:461–467. doi:10.1089/vbz.2019.2529. 32155395

[55] Kong Y, Yan C, Liu D, Jiang L, Zhang G, He B, Li Y. 2022. Phylogenetic analysis of Crimean-Congo hemorrhagic fever virus in Inner Mongolia, China. Ticks Tick Borne Dis 13:101856. doi:10.1016/j.ttbdis.2021.101856. 34763306

[56] Harvey E, Rose K, Eden JS, Lo N, Abeyasuriya T, Shi M, Doggett SL, Holmes EC. 2019. Extensive diversity of RNA viruses in Australian ticks. J Virol 93. doi:10.1128/JVI.01358-18. PMC6340049 30404810

[57] Yun Y, Heo ST, Kim G, Hewson R, Kim H, Park D, Cho NH, Oh WS, Ryu SY, Kwon KT, Medlock JM, Lee KH. 2015. Phylogenetic analysis of severe fever with thrombocytopenia syndrome virus in South Korea and migratory bird routes between China, South Korea, and Japan. Am J Trop Med Hyg 93:468–474. doi:10.4269/ajtmh.15-0047. 26033016 PMC4559681

[58] Shi J, Hu S, Liu X, Yang J, Liu D, Wu L, Wang H, Hu Z, Deng F, Shen S. 2017. Migration, recombination, and reassortment are involved in the evolution of severe fever with thrombocytopenia syndrome bunyavirus. Infect Genet Evol 47:109–117. doi:10.1016/j.meegid.2016.11.015. 27884653

[59] Wu X, Li M, Zhang Y, Liang B, Shi J, Fang Y, Su Z, Li M, Zhang W, Xu L, Wang J, Wu Q, Tang S, Wang H, Zhang T, Peng C, Zheng X, Deng F, Shen S. 2020. Novel SFTSV phylogeny reveals new reassortment events and migration routes. Virol Sin 36:300–310. doi:10.1007/s12250-020-00289-0. 32960400 PMC8087752

[60] Goodpasture HC, Poland JD, Francy DB, Bowen GS, Horn KA. 1978. Colorado tick fever: clinical, epidemiologic, and laboratory aspects of 228 cases in Colorado in 1973–1974. Ann Intern Med 88:303–310. doi:10.7326/0003-4819-88-3-303. 204240

[61] Weiss S, Dabrowski PW, Kurth A, Leendertz SAJ, Leendertz FH. 2017. A novel Coltivirus-related virus isolated from free-tailed bats from Cote d'Ivoire is able to infect human cells in vitro. Virol J 14:181. doi:10.1186/s12985-017-0843-0. 28923111 PMC5604424

[62] Liu L, Cheng J, Fu Y, Liu H, Jiang D, Xie J. 2017. New insights into reovirus evolution: implications from a newly characterized mycoreovirus. J Gen Virol 98:1132–1141. doi:10.1099/jgv.0.000752. 28548042

[63] Hisano S, Zhang R, Faruk MI, Kondo H, Suzuki N. 2018. A neo-virus lifestyle exhibited by a (+)ssRNA virus hosted in an unrelated dsRNA virus: taxonomic and evolutionary considerations. Virus Res 244:75–83. doi:10.1016/j.virusres.2017.11.006. 29122644

[64] Zahler M, Gothe R. 1997. Evidence for the reproductive isolation of Dermacentor marginatus and Dermacentor reticulatus (Acari: Ixodidae) ticks based on cross-breeding, morphology and molecular studies. Exp Appl Acarol 21:685–696. 9363622

[65] Apanaskevich DA, Horak IG. 2010. The genus Hyalomma. XI. Redescription of all parasitic stages of H. (Euhyalomma) asiaticum (Acari: Ixodidae) and notes on its biology. Exp Appl Acarol 52:207–220. doi:10.1007/s10493-010-9361-0. 20383566

[66] Gou H, Xue H, Yin H, Luo J, Sun X. 2018. Molecular characterization of hard ticks by cytochrome c oxidase subunit 1 sequences. Korean J Parasitol 56:583–588. doi:10.3347/kjp.2018.56.6.583. 30630279 PMC6327197

[67] Bolger AM, Lohse M, Usadel B. 2014. Trimmomatic: a flexible trimmer for Illumina sequence data. Bioinformatics 30:2114–2120. doi:10.1093/bioinformatics/btu170. 24695404 PMC4103590

[68] Grabherr MG, Haas BJ, Yassour M, Levin JZ, Thompson DA, Amit I, Adiconis X, Fan L, Raychowdhury R, Zeng Q, Chen Z, Mauceli E, Hacohen N, Gnirke A, Rhind N, di Palma F, Birren BW, Nusbaum C, Lindblad-Toh K, Friedman N, Regev A. 2011. Full-length transcriptome assembly from RNA-Seq data without a reference genome. Nat Biotechnol 29:644–652. doi:10.1038/nbt.1883. 21572440 PMC3571712

[69] Buchfink B, Xie C, Huson DH. 2015. Fast and sensitive protein alignment using DIAMOND. Nat Methods 12:59–60. doi:10.1038/nmeth.3176. 25402007

[70] Camacho C, Coulouris G, Avagyan V, Ma N, Papadopoulos J, Bealer K, Madden TL. 2009. BLAST+: architecture and applications. BMC Bioinformatics 10:421. doi:10.1186/1471-2105-10-421. 20003500 PMC2803857

[71] Chang WI, Lawler ELJA. 1994. Sublinear approximate string matching and biological applications. Algorithmica 12:327–344. doi:10.1007/BF01185431.

[72] Clewley JP. 1995. Macintosh sequence analysis software DNAStar's LaserGene. Mol Biotechnol 3:221–224. doi:10.1007/BF02789332. 7552691

[73] Langmead B, Salzberg SL. 2012. Fast gapped-read alignment with Bowtie 2. Nat Methods 9:357–359. doi:10.1038/nmeth.1923. 22388286 PMC3322381

[74] Kearse M, Moir R, Wilson A, Stones-Havas S, Cheung M, Sturrock S, Buxton S, Cooper A, Markowitz S, Duran C, Thierer T, Ashton B, Meintjes P, Drummond A. 2012. Geneious Basic: an integrated and extendable desktop software platform for the organization and analysis of sequence data. Bioinformatics 28:1647–1649. doi:10.1093/bioinformatics/bts199. 22543367 PMC3371832

[75] Wheeler DL, Church DM, Federhen S, Lash AE, Madden TL, Pontius JU, Schuler GD, Schriml LM, Sequeira E, Tatusova TA, Wagner L. 2003. Database resources of the National Center for Biotechnology. Nucleic Acids Res 31:28–33. doi:10.1093/nar/gkg033. 12519941 PMC165480

[76] Katoh K, Standley DM. 2013. MAFFT multiple sequence alignment software version 7: improvements in performance and usability. Mol Biol Evol 30:772–780. doi:10.1093/molbev/mst010. 23329690 PMC3603318

[77] Minh BQ, Nguyen MA, von Haeseler A. 2013. Ultrafast approximation for phylogenetic bootstrap. Mol Biol Evol 30:1188–1195. doi:10.1093/molbev/mst024. 23418397 PMC3670741

[78] Nguyen LT, Schmidt HA, von Haeseler A, Minh BQ. 2015. IQ-TREE: a fast and effective stochastic algorithm for estimating maximum-likelihood phylogenies. Mol Biol Evol 32:268–274. doi:10.1093/molbev/msu300. 25371430 PMC4271533

[79] Kalyaanamoorthy S, Minh BQ, Wong TKF, von Haeseler A, Jermiin LS. 2017. ModelFinder: fast model selection for accurate phylogenetic estimates. Nat Methods 14:587–589. doi:10.1038/nmeth.4285. 28481363 PMC5453245

[80] Muhire BM, Varsani A, Martin DP. 2014. SDT: a virus classification tool based on pairwise sequence alignment and identity calculation. PLoS One 9:e108277. doi:10.1371/journal.pone.0108277. 25259891 PMC4178126

[81] Lagkouvardos I, Fischer S, Kumar N, Clavel T. 2017. Rhea: a transparent and modular R pipeline for microbial profiling based on 16S rRNA gene amplicons. PeerJ 5:e2836. doi:10.7717/peerj.2836. 28097056 PMC5234437

