# Supplementary material for: Metatranscriptomics Reveals the Diversity of the Tick Virome in Northwest China
Source: Microbiol Spectr. 2022 Oct 10;10(5):e01115-22. doi: 10.1128/spectrum.01115-22 (PMC9602664; doi:10.1128/spectrum.01115-22)
Supplement: Supplemental file 1 — Fig S1. Download spectrum.01115-22-s0001.pdf, PDF file, 0.1 MB [file spectrum.01115-22-s0001.pdf]

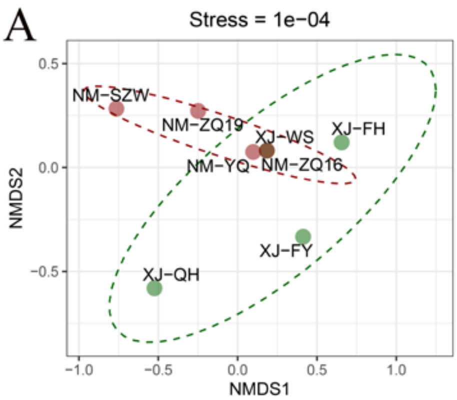

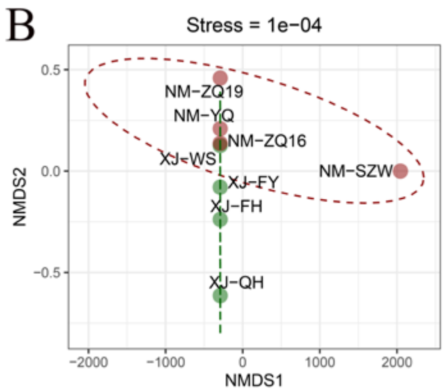

Fig S1. Non-metric multidimensional scaling (NMDS) analysis diagram of each tick library at the level of virusfamily and genus. A. NMDS of tick libraries at the level of virus family. B. NMDS of tick libraries at the level of virus genus.
